# Understanding microglial diversity and implications for neuronal function in health and disease

**DOI:** 10.1002/dneu.22777

**Published:** 2020-08-24

**Authors:** Sebastiaan De Schepper, Gerard Crowley, Soyon Hong

**Affiliations:** ^1^ UK Dementia Research Institute University College London London UK

**Keywords:** Alzheimer's disease, microglia, neuro‐immune crosstalk, synapse, transcriptional heterogeneity

## Abstract

Genetic data implicate microglia as central players in brain health and disease, urging the need to better understand what microglia do in the brain. Microglia are critical partners in neuronal wiring and function during development and disease. Emerging literature suggests that microglia have diverse functional roles, raising the intriguing question of which functions of microglia become impaired in disease to undermine proper neuronal function. It is also becoming increasingly clear that microglia exist in heterogeneous cell states. Microglial cell states appear context‐dependent, that is, age, sex, location, and health of their microenvironment; these are further influenced by external signaling factors including gut microbiota and lipid metabolites. These data altogether suggest that microglia exist in functional clusters that impact, and are impacted by, surrounding neuronal microenvironment. However, we still lack understanding of how we can translate microglia cell states into function. Here, we summarize the state‐of‐the‐art on the diverse functions of microglia in relation to neuronal health. Then, we discuss heterogeneity during developing, healthy adult and diseased brains, and whether this may be predetermined by origin and/or regulated by local milieu. Finally, we propose that it is critical to gain high‐resolution functional discernment into microglia‐neuron interactions while preserving the spatial architecture of the tissue. Such insight will reveal specific targets for biomarker and therapeutic development toward microglia‐neuron crosstalk in disease.

## 1 INTRODUCTION

The brain is the most complex, yet, highly organized, organ in our body. Therefore, it is conceivable that microglia, as tissue‐resident macrophages of the brain, exist in particular cell states that reflect the postcode of their residence and which neurons they interact with. Genetic and functional studies in multiple neurologic diseases implicate microglia to play central roles in the clearance and surveillance of their neuronal surroundings, and also in the proper maintenance and homeostasis of synaptic health and function. Recent single‐cell sequencing and proteomic studies collectively suggest microglia to exist in multiple cell states, raising the intriguing question of whether microglia exist in diverse functional states. However, we still do not know the full extent of microglial heterogeneity, and how to translate the transcriptomic cell states to function. Here, we summarize what we currently know regarding microglial diversity at both functional and transcriptomic levels. First, we review the state‐of‐the‐art on microglial function and diversity, in particular their interdependence on the neuronal microenvironment. Then, we review the current knowledge on microglial transcriptional heterogeneity in relation to functions relevant to microglia‐neuron crosstalk. We then discuss how microglial diversity is defined by various factors including origin, local milieu, and impact of peripheral immune signaling. Finally, we highlight current and future directions that we think are critical to gain insight into microglia‐neuron interactions. We propose that microglial cell states should be examined through a high‐resolution spatiotemporal lens, in a manner similar to how we examine neuronal diversity.

## 2 UNDERSTANDING HOW MICROGLIA IMPACT NEURONAL HOMEOSTASIS AND FUNCTION

**FIGURE 1:**
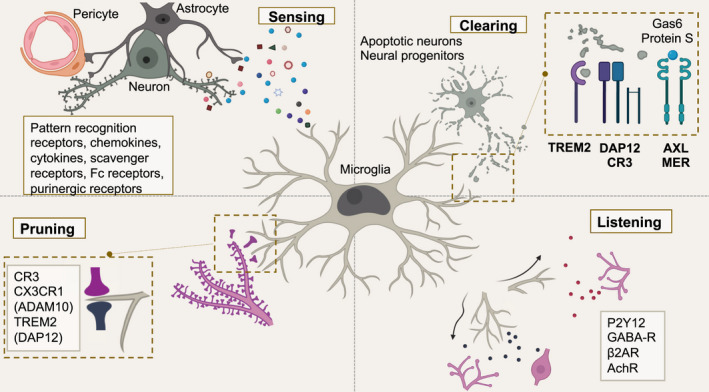
Microglia impact neuronal homeostasis and function. Figure illustrating microglia‐neuron crosstalk in the CNS. Microglia sense their neural environment by proteins encoded by sensome genes, crucial to maintain CNS homeostasis, and rapidly respond to damage or insult. Once excess neural progenitors or debris are detected, microglia initiate their clearing functions via TREM2, CR3 (DAP12), AXL, MER, and other yet‐to‐be‐defined molecules. Additionally, microglia sculpt and refine neural circuits by pruning synapses during specific developmental windows through the complement signaling pathway, fractalkine/ADAM10 signaling, and TREM2‐mediated pathways. Microglia also actively “listen” to adjacent neuronal activity by P2Y12, GABA‐R, β2AR, AchR, and other neurotransmitter receptors

Microglia are indispensable for brain wiring. They sculpt and refine neural circuits and influence synaptic development and function. However, remarkably little is known about functional states microglia assume to ensure neuronal homeostasis. Recent genetic and functional studies implicate microglia to play central roles in multiple neurologic diseases, urging the need to better understand microglia‐neuron interactions at the cellular and circuit levels. Here, we briefly review proposed roles of microglia important for neuronal homeostasis and function (Figure 1). We emphasize the importance of location and temporal window, and discuss how these functions could go awry in neurodegenerative diseases.

### 2.1 Microglia as monitors of neuronal activity

In vivo live imaging of microglia in healthy adult cortex shows highly active processes constantly surveying their niche (Davalos et al., 2005; Nimmerjahn, Kirchhoff, & Helmchen, 2005). Multiple studies have shown that this process motility is dependent on changes in neuronal activity (Cserép et al., 2020; Liu et al., 2019; Stowell et al., 2019; Wake, Moorhouse, Jinno, Kohsaka, & Nabekura, 2009). In line, maintaining constant surveillance of the neuronal microenvironment requires significant energy expenditure and microglia display remarkable metabolic flexibility to perform their sensing function (Bernier et al., 2020). The process directionality does not appear to be random. In steady‐state, microglia directly contact neuronal synapses for about 5 min every hour (Wakeet al., 2009). A recent elegant study using super resolution imaging in mice showed that microglial processes largely interact with neuronal somata versus synapses (Cserép et al., 2020). An intriguing question is whether this microglia‐neuron contact directionality shifts toward synapses during critical windows of developmental pruning and during aberrant synapse loss in disease. Indeed, in acute laser‐induced injury, microglial processes rapidly converge to target the site of injury (Davalos et al., 2005). The target‐oriented form of motility is mediated by purinergic signaling, that is, activity/injury‐induced ATP and ADP and P2Y12 receptor on microglia. In microglia‐neuron somatic junctions, microglial P2Y12 directly interacts at potassium channel Kv2.1 clusters on neurons (Cserép et al., 2020).

Microglia‐neuron crosstalk is also mediated through cytokines, neurotransmitters, and neuropeptides. For example, microglia produce neuropeptides and growth factors. An elegant study in healthy adult mice showed that microglia in motor cortex contribute to learning‐dependent spine formation via microglial brain‐derived neurotrophic factor (BDNF) (Parkhurst et al., 2013). It remains unclear what regulates microglial BDNF in this paradigm. In a model of peripheral nerve injury, ATP‐stimulated microglia release BDNF and induce neuronal hyperexcitability by inverting the polarity of neuronal GABA currents (Coull et al., 2005). Further, microglial insulin‐like growth factor‐1 (IGF‐1) promotes survival of neural progenitors in layer V cortical neurons (Parkhurst et al., 2013; Ueno et al., 2013; Ziv et al., 2006). The microglial P2Y12 receptor, but not fractalkine receptor (CX3CR1), has been shown to be critical in neuronal activity‐dependent synaptic plasticity in visual cortex (Lowery, Tremblay, Hopkins, & Majewska, 2017; Schecter et al., 2017; Sipe et al., 2016). P2Y12 signaling also dictates microglial responses during neuronal NMDA receptor activation (Dissing‐Olesen et al., 2014; Eyo et al., 2014), neuropathic pain (Gu et al., 2016) and ischemia (Cserép et al., 2020).

Certain glia‐derived factors such as TNFα and IL‐1β have been reported to impact synaptic activity. For example, TNFα regulates synaptic scaling, a mechanism that allows neurons to stabilize excitatory and inhibitory synaptic weights in response to prolonged periods of reduced activity (Stellwagen & Malenka, 2006). Also, hippocampal IL‐1β has been shown to be necessary for fear‐conditioned memory (Goshen et al., 2007; Rogers et al., 2011). Further, several studies have suggested that microglia express receptors to sense changes in neurotransmitter and neuropeptide concentrations, including metabotropic glutamate receptors (Biber et al., 1999; Taylor, Diemel, Cuzner, & Pocock, 2002; Taylor, Diemel, & Pocock, 2003), GABA_A_ receptors (Lee, Schwab, & McGeer, 2010), GABA_B_ receptors (Kuhn et al., 2004), adrenergic receptors (Färber, Pannasch, & Kettenmann, 2005; Mori et al., 2002; Tanaka, Kashima, Suzuki, Ono, & Sawada, 2002), and acetylcholine receptors (Shytle et al., 2004; Suzuki et al., 2006), among many others. Further, activation of these receptors modulates microglial cytokine release (comprehensively reviewed in York, Bernier, & MacVicar, 2018), including TNFα and IL‐6 in cell culture (Lee et al., 2010; Mori et al., 2002; Shytle et al., 2004) and brain slices (Färber et al., 2005). Interestingly, heterogeneous subpopulations of microglia may exist that express distinct sets of receptors in vivo, especially in regard to age, thus displaying a varying degree of response to a given neurotransmitter or neuropeptide (Pannell et al., 2016; Pannell, Szulzewsky, Matyash, Wolf, & Kettenmann, 2014; Seifert, Pannell, Uckert, Färber, & Kettenmann, 2011). Altogether, changes in neurotransmitters in the microenvironment could stimulate microglia to release inflammatory cytokines and chemokines, thereby adversely impacting nearby neuronal networks. Overall, it is clear that microglia engage multiple neuronal signaling mechanisms, highlighting their role as facilitators of neuronal function in the central nervous system (CNS).

### 2.2 Microglia as sculptors of neuronal synapses

Microglia enter the CNS around embryonic day 9, making them ideal protagonists in sculpting brain circuitry (Ginhoux et al., 2010). Indeed, alteration in microglial genes leads to sustained defects in brain wiring (for in‐depth review, see Wilton, Dissing‐Olesen, & Stevens, 2019). It is important to note that synaptic pruning during development is highly regulated in a spatiotemporal manner (Boulanger & Shatz, 2004; Hua & Smith,^2004^). Further, neural circuit refinement is dependent on neuronal activity. It is now becoming increasingly clear that microglia crucially contribute to this activity‐dependent refinement (for in‐depth review, see Neniskyte & Gross, 2017). Several pathways have been implicated in microglia‐mediated synaptic pruning. One critical pruning mechanism that has been studied in the visual system is the classical complement cascade, a highly conserved innate immune pathway that mediates the removal of opsonized debris or pathogens (Gasque, 2004). In the developing visual thalamus, C1q, C3, and CR3 mediate synaptic pruning of retinal ganglion axons (Schafer et al., 2012; Stevens et al., 2007). Interestingly, complement (CR3) does not appear to play a role in developmental synaptic pruning in the hippocampal CA1 stratum radiatum (Weinhard et al., 2018). Whether complement‐mediated synaptic pruning pathway in microglia is involved in developmental neural circuit refinement of other brain regions is yet to be determined. In the postnatal developing hippocampal CA1, the fractalkine signaling pathway (CX3CR1‐CX3CL1) plays a critical role in circuit refinement (Paolicelli et al., 2011; Zhan et al., 2014). It was suggested that microglia contribute to circuit refinement in the developing hippocampus by phagocytosing spines (Paolicelli et al., 2011); however, a follow‐up study using correlative light and electron microscopy (CLEM) in slice cultures showed lack of direct evidence for spine phagocytosis (Weinhard et al., 2018). Further experiments are needed to decipher how microglial CX3CR1 mediates neural circuit refinement in the developing hippocampus. The fractalkine neuroimmune axis, in conjunction with ADAM10, has been shown to be critically involved in microglial engulfment of barrel cortex synaptic inputs after lesioning of mouse whiskers (Gunner et al., 2019). Finally, a recent elegant study showed that TREM2, which is exclusively expressed on myeloid cells and microglia in the CNS (Kiialainen, Hovanes, Paloneva, Kopra, & Peltonen, 2005; Schmid et al., 2002), also plays a role in developmental synapse pruning in the hippocampus, the manipulation of which results in sustained deficits in social behavior (Filipello et al., 2018). Further, mice expressing mutations in DAP12, an adaptor protein for TREM2 signaling, display impaired synaptic maturation (Roumier et al., 2004), suggesting that TREM2‐DAP12 signaling plays an integral role in circuit refinement.

Importantly, microglial engulfment of synapses is activity‐dependent (Gunner et al., 2019; Schafer et al., 2012; Tremblay, Lowery, & Majewska, 2010). Microglia appear to selectively engulf the less active synapses (Schafer et al., 2012), raising the intriguing question of how microglia discern which synapses to engulf. In postnatal organotypic hippocampal slice cultures, and in the absence of additional injury or damage, CLEM showed that microglia engulf synaptic structures through “nibbling,” termed trogocytosis (Weinhard et al., 2018). It will be critical to investigate these microglia‐synapse interactions in vivo using high‐resolution time‐lapse imaging, and how microglia‐neuron interactions shift upon changes in neuronal activity or during disease. Molecularly, there appears to be a balance of “don't‐eat‐me” and “eat‐me” signals on synapses (Lehrman et al., 2018; Rivest, 2018). Indeed, recent data propose CD47/SIRPα as a “don't‐eat‐me” signal regulating microglia‐synapse pruning (Lehrman et al., 2018). Further, exposure of phosphatidylserine on the outer leaflet of membranes is emerging as a vital “eat‐me” signal (Païdassi et al., 2008) on synapses (Györffy et al., 2018; Li et al., 2020) (Scott‐Hewitt EMBO [accepted] 2020). Further experiments are needed to decipher how neuronal activity modulates expression of these signals. Astrocytes also appear to play critical roles; they have been shown to engulf both excitatory and inhibitory synapses in visual thalamus via MEGF10 and MERTK (Chung et al., 2013). Astrocytes also work together with microglia via IL‐33 signaling in spinal cord and thalamus (Vainchtein et al., 2018). Neuronal IL‐33 in the adult hippocampus acts on microglia to remodel the extracellular matrix, allowing enhanced dendritic spine plasticity with effects on fear memory (Nguyen et al., 2020). Finally, it is important to note that some immune pathways that regulate synaptic pruning do not involve microglia. A key example is the major histocompatibility complex I‐paired immunoglobulin‐like receptor B (MHCI‐PirB) pathway (Datwani et al., 2009; Kim et al., 2013; Lee et al., 2014). Both molecules are expressed by neurons, are regulated by neuronal activity, and have been shown to be necessary and sufficient for synaptic elimination in the developing visual system as well as during disease (Kim et al., 2013; Lee et al., 2014; William et al., 2012).

Importantly, embryonic microglial depletion perturbs the inhibitory wiring provided by parvalbumin‐expressing interneurons in the mouse barrel cortex (Thion et al., 2019), suggesting modulation of both excitatory and inhibitory circuitry by microglia. These data altogether suggest that synaptic pruning involves multiple cell types and depending on the circuit, time and brain region, distinct pathways are employed.

### 2.3 Microglia as local phagocytes and sensors of neuronal environment

As tissue‐resident macrophages of the brain parenchyma, microglia phagocytose apoptotic neurons during development (Parnaik, Raff, & Scholes, 2000) as well as progenitors in the hippocampus (Diaz‐Aparicio et al., 2019; Sierra et al., 2015) and olfactory bulb (Wallace, Lord, Dissing‐Olesen, Stevens, & Murthy, 2020) in adult steady‐state brains. A key mechanism by which microglia phagocytose apoptotic neurons is by *Mer* and *Axl* (Fourgeaud et al., 2016). Mice that lack MER and AXL specifically in microglia accumulate neuronal progenitor cells in the subventricular zone, and this phagocytotic process seems to driven by TAM receptor ligands Gas6 and Protein S (Fourgeaud et al., 2016). Importantly, microglial phagocytosis concurs with the upregulation of a unique neurogenic secretome (i.e., VGF, VEGF, FGF2), potentially suggesting a feedback loop for neurogenesis (Diaz‐Aparicio et al., 2020; Elmadany et al., 2020). Alternative to phagocytosis, hippocampal microglia contribute to neuronal cell death via CR3‐DAP12‐dependent production of superoxide ions (Wakselman et al., 2008). Altogether, these examples demonstrate the essential role of microglia in remodeling neural stem cells within different niches of the brain.

Further, microglia act as the primary damage sensors of the CNS. Microglia use a unique sensome that consists of around 100 genes that encode for pattern recognition receptors (*Tlr2, Tlr7, Siglec‐H*), chemokine receptors (*Ccr3*, *Cx3cr1, Cxcr2, Cxcr4*), Fc receptors (*Fcgr1*, *Fcgr3, Fcg2b*), purinergic receptors (*P2rx7, P2rx4*), cytokine receptors (*Ccr5*), and a broad array of scavenger receptors (*Cd36, Marco*) (Areschoug & Gordon, 2009; Hickman et al., 2013). Of note, microglial sensome genes are uniformly expressed across brain regions, suggesting that microglia are ubiquitously equipped to perform their sensing function. Microglia also use TREM2‐DAP12 signaling to sense damage‐associated lipids (Wang et al., 2015). Soluble forms of TREM2 produced by proteolytic cleavage regulate phagocytosis and expression of pro‐inflammatory cytokines (Zhong & Chen, 2019). Moreover, soluble TREM2 is increased in cerebrospinal fluid of patients with autosomal dominant AD, implicating its biomarker potential for microglia activation (Suarez‐Calvet et al., 2016). Strikingly, microglia in aged mice shift their sensome toward increased expression of pro‐inflammatory chemokines such as *Ccl4, Ccl3, Ccl2*, *Ccl12,* and *Cxcl12*, suggesting enhanced chemotaxis of monocytes, lymphocytes, and other immune cells (Hickman et al., 2013).

In all these situations, microglia interact with other glial cells including astrocytes (Lee et al., 2010; Liddelow et al., 2017; Skripuletz et al., 2012; Tanuma, Sakuma, Sasaki, & Matsumoto, 2006; Vainchtein et al., 2018; Yu et al., 2019), oligodendrocytes (Cantuti‐Castelvetri et al., 2018; Lloyd et al., 2019; Lloyd & Miron, 2019; Ransohoff, Hafler, & Lucchinetti, 2015; Safaiyan et al., 2016), and pericytes (Attwell, Mishra, Hall, O’Farrell, & Dalkara, 2016; Giannoni et al., 2018; Lendahl, Nilsson, & Betsholtz, 2019; Matsumoto et al., 2018; Nortley et al., 2019). These crosstalks are functionally important but outside the scope of this review; for insights, please refer to: Jha, Jo, Kim, and Suk (2019); Vainchtein and Molofsky (2020); Lloyd and Miron (2019) and Rustenhoven, Jansson, Smyth, and Dragunow (2017). Finally, we need to better understand how microglia communicate with other immune cells of the CNS, including border‐associated macrophages (BAMs) and adaptive immune cells (Kierdorf, Masuda, Jordão, & Prinz, 2019; Korin et al., 2017). Indeed, given recent revelations on the brain's waste pathway including the glymphatic system and the elaborate network of functional lymphatic vessels throughout the brain (Aspelund et al., 2015; Louveau et al., 2015; Lukić, Gluncić, Ivkić, Hubenstorf, & Marusić, 2003; Mesquita, et al., 2018), it will be critical to understand how microglia and BAMs get rid of their waste and work with non‐macrophage immune cells circulating in the lymphatic network to maintain brain homeostasis (Da Mesquita, Fu, & Kipnis, 2018; Mestre, Mori, & Nedergaard, 2020).

### 2.4 Deciphering which functions of microglia fail in disease

Microglia are increasingly recognized as central players in neurologic diseases. Of note, the use of epilepsy models has uncovered multiple signaling pathways contributing to neuron‐microglia communication during seizure activity, including P2Y12 (Mo et al., 2019), CCL2‐CCR2 (Tian et al., 2017), and CX3CL1 (Eyo et al., 2017). In Alzheimer's disease (AD), there is a strong genetic rationale for immune dysfunction to increase risk for dementia (e.g., *CR1*, *MS4A*, *PLCG2*, *ABI3,* and *TREM2*) (Efthymiou & Goate, 2017; Guerreiro, Bras, & Hardy, 2013; Jansen et al., 2019; Kunkle et al., 2019). Key questions now are to decipher how these mutations in microglia impair microglia‐neuron crosstalk to facilitate neuronal loss and dysfunction, and whether we can identify the microglia that have gone awry in neurodegeneration. For instance, EM analysis of microglia in the diseased brain revealed a so‐called “dark microglia,” which has electron‐dense cytoplasm and nucleoplasm under oxidative stress during neurodegeneration (Bisht et al., 2016). Recent data using animal models and patients are helping us understand how microglia that carry mutations in risk genes or loci contribute to major AD pathological hallmarks, including amyloid plaque deposition, maintenance and clearance (Andrews, Fulton‐Howard, & Goate, 2020). Some of these studies have also highlighted major differences between human and mouse microglia (Geirsdottir et al., 2019; Mancuso et al., 2019; Sala Frigerio et al., 2019; Zhou et al., 2020), raising the importance of considering species‐specific differences when investigating neuroimmune interactions. One function of microglia that likely malfunctions in AD is their ability to sense damage. *Trem2*, a key AD risk gene (Guerreiro, Wojtas, et al., 2013; Jonsson et al., 2013), appears to be critical for this “sensing” ability and downstream damage response. Mice with defective TREM2 signaling display impaired microglial response to injury and amyloid plaque pathology (Kleinberger et al., 2017; Ulland et al., 2017; Wang et al., 2015), a phenotype also demonstrated in human AD brain tissue (Toomey et al., 2020; Ulrich et al., 2014; Wang et al., 2016) (for a recent review on TREM2, please refer to: Deczkowska, Weiner, & Amit, 2020). Further, TREM2 is vital for sensing damaged lipids (Wang et al., 2015), maintaining proper lipid homeostasis (Jaitin et al., 2019; Nugent et al., 2020) and sustaining energy metabolism (Ulland et al., 2017). An intriguing question is whether these microglia also fail to monitor neighboring neuronal health and function. In support of this, loss‐of‐function mutations in *TREM2* or *DAP12* underlie Nasu–Hakola disease, where patients display progressive presenile dementia (Paloneva et al., 2000, 2002). Moreover, as mentioned above, TREM2 has recently been shown to play a role in microglia‐mediated synaptic refinement in brain development (Filipello et al., 2018). Further studies are warranted to establish the link between TREM2 and synaptic impairment in AD. Another major function of microglia that become dysregulated early in AD is their engulfing of synapses. In AD mouse models, prior to plaque‐related neuroinflammation but when synapses are already vulnerable (Selkoe, 2002; Wyss‐Coray & Rogers, 2012), the synaptic pruning pathway involving the classical complement cascade (C1q, C3, CR3) is reactivated in a region‐specific manner (Dejanovic et al., 2018; Hong et al., 2016; Paolicelli et al., 2017; Shi et al., 2017; Wu et al., 2019). Now, a similar reactivation of the complement pathway in microglia and relevance to synapse loss has been reported in various models of neurologic diseases (Dejanovic et al., 2018; Hong et al., 2016; Lui et al., 2016; Paolicelli et al., 2017; Sellgren et al., 2019; Shi et al., 2017; Vasek et al., 2016; Vukojicic et al., 2019; Werneburg et al., 2020; Wu et al., 2019), implicating the microglial pruning pathway as a potential common therapeutic target across diseases.

As we gain deeper insight into microglial functional states, we will be able to better elucidate how, and which, microglia become dysfunctional at various stages of the disease. This will enable dissection of how microglia contribute to brain dyshomeostasis and gain insight into specific pathways worth targeting to preserve synapses and neuronal function.

## 3 LINKING MICROGLIAL CELL STATES TO FUNCTION RELEVANT TO NEURON‐MICROGLIA CROSSTALK

**FIGURE 2:**
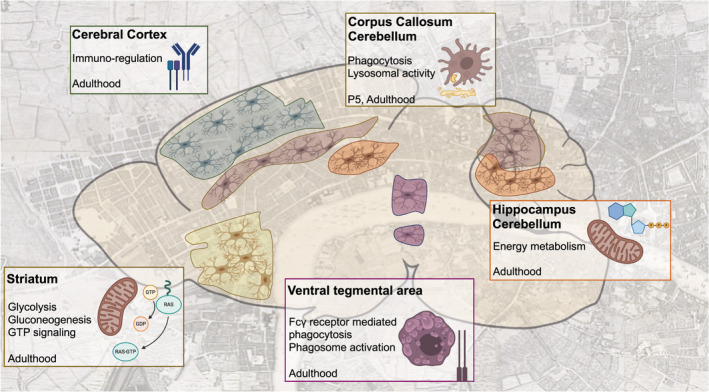
Microglia cell states in adulthood: does postcode matter? Transcriptomic analyses of microglia isolated from different brain regions support the existence of region‐specific functional cell states. Dependent on their local microenvironment or “*residential* *postcode*,” microglia acquire functional phenotypes that support local neuronal development and function. GO biological processes are based on enriched transcripts of microglia residing in that particular region (Ayata et al., 2018; De Biase et al., 2017; Grabert et al., 2016)

Traditionally, microglia have been defined by morphology, ontogeny, density, or “activation” profiles. The recent advent of single‐cell RNA sequencing (scRNA seq) has revealed a high degree of transcriptional heterogeneity that reflects the dynamic CNS microenvironment in space and time. However, to fully define microglial identity, we need to integrate its transcriptional profile with cellular function. Here, we briefly review the state‐of‐the‐art on microglial transcriptional cell states but with an emphasis on the functional clusters they may represent. Further, we highlight here the need to consider the spatiotemporal axis when evaluating microglial cell states and function. We propose that microglia should be defined in their native spatial location or “*residential postcodes*” (Figure 2).

### 3.1 Hats microglia wear as brain develops and ages

A key hallmark of tissue‐resident macrophages is their unique plasticity to adapt to functional demands of the tissue in which these cells reside (Gautier et al., 2012; Okabe & Medzhitov, 2014). Accordingly, it is assumed that microglia are most heterogenous during early development, reflecting dynamic periods of neurogenesis and synaptic remodeling (Hammond et al., 2019; Li et al., 2019; Masuda et al., 2019). scRNA seq studies showed at least six major subclasses of microglia during early development, but without appreciable sex differences (Hammond et al., 2019; Masuda et al., 2019). In contrast, sex differences impact adult microglia as demonstrated by the higher antigen‐presenting capacity in male microglia (Guneykaya et al., 2018). Interestingly, one cluster of postnatal microglia, the axon tract‐associated microglia (ATM), appears on unmyelinated axon tracts in corpus callosum and cerebellum around P4/P5 (Hammond et al., 2019). The ATM show enriched expression transcripts involved in lipoprotein metabolism (*Apoe, Soat1, Lpl*), lysosomal activity (*Ctsd, Ctsl, Ctsb*), and phagosome formation (*Cyba, Clec7a*) (Hammond et al., 2019). These enriched transcripts are possibly a reflection of functionally specialized microglia that are actively refining neuronal circuits, taking into account that lipids are the main components of myelin sheets, synapses, and dendrites. A comparable population was characterized by a second scRNA seq study, showing an intimate interaction of ameboid *Spp1* ^+^ *Igf1^+^Clec7a^+^* microglia with lipid‐rich oligodendrocytes in the white matter (Li et al., 2019). These data altogether suggest that diverse functional states exist within the white matter, and that these microglial subpopulations may exercise a division of labor. Of note, many of the transcripts expressed by these putative phagocytosing microglia in the white matter are also expressed by disease‐associated microglia (DAM) that surround amyloid plaques in AD mouse models (Keren‐Shaul et al., 2017; Li et al., 2019). Furthermore, outside the CNS, highly similar gene profiles are found in lipid‐associated macrophages within adipose tissues, as well as in aortic macrophages during atherosclerosis that is characterized by the accumulation of extracellular lipids (Bobryshev, Ivanova, Chistiakov, Nikiforov, & Orekhov, 2016; Jaitin et al., 2019). This could point toward a conserved response of myeloid cells across different tissues toward a set of environmental cues that are shared in development and neurodegenerative disease.

During aging, microglia are altered in a region‐specific manner, as demonstrated by the selective upregulation of transcripts involved in cell adhesion and motility in hippocampal microglia, but not in cerebellar microglia, over time (Grabert et al., 2016). This phenotypic shift is consistent with the expansion of certain clusters that were found at very low levels during adulthood (Hammond et al., 2019; Sala Frigerio et al., 2019; Sankowski et al., 2019). A more in‐depth comparison between microglia from adult (P100) and aged (P540) mice highlighted that these two age‐associated clusters were enriched for inflammatory—(*Ccl4, Ccl3, Il1b, Cst7*) and interferon‐related (*Ifitm3, Rtp4, Oasl2*) genes respectively, suggesting their possible involvement in age‐related CNS inflammation (Hammond et al., 2019). Moreover, in this study, two other clusters highlight the presence of monocytes and macrophages, indicating the coexistence of ontologically distinct myeloid cells in aged brains (Hammond et al., 2019). It will be interesting to understand the functional implications of such intermixed populations in the aging or injured brain.

### 3.2 Residence postcode may define microglial cell states

Following development, adult microglia integrate divergent cellular states tied to the brain region of residence (Figure 2). Importantly, scRNA seq of isolated brain regions identified 32 subclusters among telencephalon‐projecting neurons in areas such as cortex, hippocampus, and striatum (Zeisel et al., 2018). As such, neuronal diversity, driven by distinct neuronal subtypes, neurotransmitters and neuropeptides, likely provide niche signals for microglial imprinting in the steady‐state adult brain. For example, cerebellar and hippocampal microglia express transcripts related to energy metabolism, contrasting cortical and striatal microglia that are enriched in immune signaling genes such as *Trem2* and *SiglecH* (Grabert et al., 2016). Further, cerebellar microglia show increased levels of CD68 and genes related to endocytosis and phagocytosis, implicating functional specialization potentially related to ongoing neuronal turnover (Ayata et al., 2018). On the contrary, transcripts in striatal microglia indicate active GTP signaling, whereas microglia in the ventral tegmental area (VTA) showed transcripts involved in Fcγ receptor‐mediated phagocytosis, phagosome maturation and growth factor signaling (Ayata et al., 2018; De Biase et al., 2017). Single‐cell analyses from isolated brain regions have further confirmed metabolic diversity among adult microglia, exemplified by subclusters with marker genes related to lysosomal pathways (*Ctsd, Lamp2*), cholesterol/lipid metabolic pathways (*Plin2, Pld4, Ptgs1*), or phagosome activation (*Ctss, Rab5c*) (Masuda et al., 2019). Considering that many brain disorders are accompanied by changes in brain energy metabolism, it would be interesting to assess metabolic profiles of microglia in vulnerable brain regions in various disease settings (Aldana, 2019). However, some scRNA seq studies in adult microglia from whole brains concluded minimal transcriptional heterogeneity in adult microglia. Sala Frigerio et al. identified only two subclusters (termed H1M and H2M) of microglia in dissected cortex and hippocampus, collectively constituting (80%–90%) of total microglia (Sala Frigerio et al., 2019). Li et al. defined only one homeostatic cluster among *Tmem119^+^* microglia isolated from different brain regions at P60, contrasting earlier reports on regional microglial heterogeneity (Grabert et al., 2016; Li et al., 2019; Masuda et al., 2019). Using deep single‐cell and bulk RNA sequencing on isolated brain regions, the authors found high correlation between microglia from different brain regions and attributed previously described regional differences to non‐microglial populations (Li et al., 2019). Nevertheless, the use of TMEM119 to sort microglia may overlook the existence microglia subpopulations expressing low levels of *Tmem119* (Bennett et al., 2016; Masuda et al., 2019). Further, cell isolation protocols induce microglial epigenetic and transcriptomic changes that occur rapidly upon tissue dissociation (Haimon et al., 2018).

Besides microglia, the CNS myeloid compartment consists of BAMs that reside at brain borders, including meninges, perivascular space, and choroid plexus (Goldmann et al., 2016; Kierdorf et al., 2019). BAMs express clear niche‐specific signature genes including *Lyve1, P2rx7* (subdural BAM), *Pla2g2d, Ccl8* (dural BAM), or *Lilra5, Ttr* (choroid plexus BAM) (Van Hove et al., 2019). Of interest, BAMs share the expression of particular genetic programs (*Lyve1, Cd209f, Cd209, Fcna*) with macrophages that reside in vasculature‐associated niches in the lung, fat and dermis, suggesting that macrophages acquire common specialized functions that are imprinted by cues from conserved cross‐tissue niches (Chakarov et al., 2019). In accordance, particular functional attributes including neuronal surveillance and neurotrophic support are not unique to microglia, but are also observed in peripheral macrophages that are associated with neuronal structures. For example, nerve‐associated macrophages are found in the myenteric plexus, part of the enteric nervous system or “little brain of the gut,” where they are involved in providing trophic support for neuronal survival (De Schepper et al., 2018). It will be interesting to investigate whether similar mechanisms of neuroimmune interaction are employed in the brain and in the gut (Verheijden, De Schepper, & Boeckxstaens, 2015).

Altogether, these data suggest that spatiotemporal organization (i.e., brain regions and age) is an important determinant of microglial heterogeneity and functional specialization. Further, microglia communicate with synapses and neuronal soma via their numerous processes, implicating that the subcellular organization of transcripts may hold important information. Taking into account that microglia likely lose processes during sampling and cell isolation protocols that precede the preparation of single‐cell suspensions, in situ sequencing techniques will be critical to decipher which cell states may be fundamental to neuronal homeostasis and function.

## 4 UNDERSTANDING WHAT DEFINES MICROGLIAL HETEROGENEITY

The existence of microglial heterogeneity raises the question of how these cells are instructed to support developmental and functional requirements of the CNS. As we continually refine our understanding of microglia ontogeny and transcriptional networks, an important goal is to determine how these cells are instructed by their origin versus environment. Microglia and BAMs are separated from the circulation via the blood‐brain barrier, so differentiation trajectories could be attributed to their CNS environment. In addition, peripheral and microbial components have been linked to the imprinting of microglial identity.

### 4.1 Role of embryonic origin

Microglia and BAMs are unique among tissue‐resident macrophages in that they are derived from primitive progenitors in the yolk‐sac and persist during adulthood, raising the important question of whether their embryonic ontogeny matters for functional diversity in the CNS (Ginhoux et al., 2010; Utz et al., 2020). Fate‐mapping studies have shown that microglia are derived from a primitive wave of erythromyeloid precursors (EMPs) that appear in the blood islands of the yolk‐sac around E7, subsequently giving rise to primitive nucleated erythrocytes and macrophages (Ginhoux et al., 2010; Palis, Robertson, Kennedy, Wall, & Keller, 1999). In contrast, most tissue‐resident macrophages outside of the CNS are derived from a second or “transient‐definitive wave” of EMPs that originate in the yolk‐sac but move to the fetal liver thereafter (Hoeffel et al., 2015). Interestingly, embryonic progenitors segregate as early as E10.5 into CD206^+^ and CD206^–^ macrophages, suggesting early lineage imprinting (Utz et al., 2020). Further, a subpopulation of *HoxB8* ^+^ microglia was found to be generated during the “late wave” of EMPs, suggesting that heterogeneity of microglia progenitors may exist prior to CNS infiltration (De et al., 2018). However, it remains unclear whether this heterogeneity of progenitors matters for functional diversity in the developing brain.

Several groups have investigated as to whether hematopoietic stem cell (HSC)—or bone marrow‐derived monocytes engrafted in the CNS could recapitulate the functional phenotype of microglia by using distinct models of microglia depletion and/or HSC‐transplantation models (Bennett et al., 2018; Cronk et al., 2018; Lund et al., 2018; Shemer et al., 2018). Although the engrafted cells exhibit a gene expression profile that is comparable to embryonic microglia, these cells still remained distinct in terms of transcriptome and chromatin states, even after extended time of adaption (6–8 months) to the neural environment. Further, embryonic microglia and engrafted cells differed in their functional response to peripheral lipopolysaccharide (LPS) stimulation, suggesting that HSC‐derived cells are not able to fully acquire the identity of preexisting embryonic microglia (Shemer et al., 2018). This indicates that the microglia‐specific gene signature, characterized by *Sall1, Gpr56, P2ry12,* and *Slc2a5* among others, could be at least partly determined by their embryonic origin. Interestingly, loss of *Sall1* in embryonic‐derived microglia induced a pro‐inflammatory phenotype and reduced proliferation of doublecortin‐positive neuroblasts in the hippocampal dentate gyrus, suggestive of decreased neurogenesis (Buttgereit et al., 2016). Further, postnatal ablation of *Gpr56* in *Cx3cr1* ^+^ macrophages resulted in higher density of synapses in stratum lacunosum moleculare of the hippocampus at postnatal week three, suggesting the importance of yolk‐sac signature genes for proper neurodevelopment (Li et al., 2020). An important note here is that microglia of embryonic origins may carry epigenetic programs (so‐called “poised” enhancers) that determine functional diversity (Amit, Winter, & Jung, 2016; Gosselin et al., 2014; Lavin et al., 2014). In this, chromatin accessibility studies at the single‐cell level may provide new insights into microglial heterogeneity and diverse functions.

### 4.2 Role of the neural environment

In addition to ontogeny, microglia and BAMs are imprinted by environmental signals derived from neighboring neural cells (Bennett et al., 2018; Gosselin et al., 2017; Van Hove et al., 2019). As such, microglia quickly lose their homeostatic signature and deactivate enhancers and transcription factor binding sites within hours after isolation, likely contributing to inconsistent observations of microglia transcriptomes as noted earlier (Gosselin et al., 2017). Microglia are dependent on expression patterns of CSF1 (in cerebellum) and IL‐34 (in forebrain), CX3CL1, transforming growth factor–β (TGF‐β), and others that are unique to the spatiotemporal context of the brain (Butovsky et al., 2013; Kana et al., 2019; Wang et al., 2012). Microglia, like other tissue‐resident macrophages, critically rely on the CSF1R‐CSF1 signaling axis, yet, *Csf1* ^op/op^ mice (carrying an inactivating mutation in the *Csf1* gene) only moderately reduce the presence of adult microglia (Ginhoux et al., 2010). In line, depletion of a super‐enhancer in the *Csf1r* locus (*Csf1r* ^ΔFIRE/ΔFIRE^) impairs differentiation of microglia (Rojo et al., 2019). In contrast to *Csf1r‐*deficient mice, *Csf1r* ^ΔFIRE/ΔFIRE^ mice do not show gross loss of neuronal progenitors, suggesting that CSF1R signaling might be redundant for brain development (Rojo et al., 2019). In fact, microglia rely at least partly on IL‐34, the alternative ligand for CSF1R that is highly expressed by neurons predominantly in the cortex, olfactory nucleus, and the hippocampus (Hickman et al., 2013; Kana et al., 2019). Consequently, genetic deletion of *Il34* versus *Csf1* has distinct effects on microglia distribution; cortical microglia numbers were unaffected by CSF1 deficiency while cerebellar microglia survival was independent of IL‐34 depletion (Kana et al., 2019). These studies further suggest that regular spacing of microglia in different brain regions could be controlled by gradients of distinct cytokines in their local milieu. A recent study demonstrated a similar interdependency for TGF‐β among CNS tissue‐resident macrophages (Utz et al., 2020). Although TGF‐β is a CNS identity signal that controls microglial maintenance (Butovsky et al., 2013), *Tgfbr2* depletion in *Vav1^+^* cells (labeling all hematopoietic progeny from E11.5 onward) does not affect BAMs (Utz et al., 2020), emphasizing the existence of at least two independent developmental pathways among CNS‐resident macrophages. It is interesting to note that BAMs fail to infiltrate and replenish empty niches in *Vav1^iCre^Tgfbr2^fl/fl^* mice (Utz et al., 2020). This suggests that other niche‐specific environmental signals regulate migration and maturation, or alternatively, that BAMs and microglia are embryonically *“hard‐wired”* or prespecified in their brain colonization patterns. Further lineage tracing of microglia and BAMs, in combination with single‐cell transcriptomics, epigenetics as well as functional studies, are needed to delineate these questions.

### 4.3 Peripheral immune signaling and microglia imprinting

Peripheral signals impact CNS development and function. Systemic inflammation during pregnancy is associated with defects in synaptic connectivity and maturation later in life (Estes & McAllister, 2016; Meyer et al., 2006; Patterson, 2009). Intriguingly, challenging pregnant dams with viral or bacterial components such as polyI:C or LPS shifts early microglia differentiation toward a more advanced developmental stage (Matcovitch‐Natan et al., 2016). In parallel, similar mouse models of maternal immune activation develop behavioral changes and neurodevelopmental defects in adult offspring, suggesting that genetic alterations in microglia during embryonic development is detrimental for proper brain development (Smith, Li, Garbett, Mirnics, & Patterson, 2007). Of interest, germ‐free mice exhibit overt defects in spine formation, paralleled by transcriptomic alterations in excitatory neurons and microglia (Chu et al., 2019). Earlier studies highlighted enrichment of interferon and sensome genes, as well as decreased chromatin accessibility in embryonic microglia isolated from germ‐free animals (Matcovitch‐Natan et al., 2016). Interestingly, microglia in male offspring displayed more transcriptional differences during embryonic development, in contrast to female microglia that exhibited many dysregulated genes linked with adaptive immune responses and chemotaxis during adulthood (Matcovitch‐Natan et al., 2016; Thion et al., 2018). These data suggest that there are sex‐differences in how maternal microbiota impact microglial development and imprinting. Importantly, administration of metabolites of gut microbiota, such as short‐chain fatty acids, was able to reverse some of the transcriptional and morphological changes in germ‐free mice and allowed microglia to acquire their homeostatic transcriptome (Erny et al., 2015). Microbiome composition has changed over evolution, suggesting that microbial diversity might contribute to the observed heterogeneity among different microglia species (Geirsdottir et al., 2019; Youngblut et al., 2019). Although microglia express a conserved core gene program of orthologous genes (*Csf1r, P2ry12*), a few species‐specific transcriptomes were observed, for example, *Fcrls* in murine microglia, or C3 and SPP1 within primates (Geirsdottir et al., 2019). Of interest, a recent article further highlighted how peripheral signaling can influence microglial diversity in different brain regions; overexpression of human TNFα resulted in increased expression of transcripts related to complement and inflammation in cortical, striatal, and thalamic microglia, but not in hippocampal nor cerebellar microglia (Süß et al., 2020). Together, further studies are necessary to explore how microglia functional diversity is influenced by signals derived from the periphery, including cytokines, metabolites, and other circulating factors.

In summary, microglial identity is determined by ontological *“hard‐wiring*” and the progressive imprinting by signals derived from the developing CNS and periphery. Combining these concepts, an intriguing, yet, unsolved question is raised: are cell states predestined or instructed upon arrival at their microenvironmental niche in the CNS? Combining fate‐mapping with in situ analyses and functional assays with single‐cell resolution will provide foundation for future development of targeted therapeutic approaches.

## 5 CONCLUSION

Over the past decade, the advent of powerful genomic and proteomic tools, along with functional studies in microglia, have significantly enhanced what we know about microglial biology and their impact on neuronal wiring and function during development, steady‐state, and disease. It is also clear that microglia display heterogeneous transcriptomic and diverse functional profiles that are likely determined by the specific brain regions they reside in and the neuronal circuits they are associated with. Critical questions arise: *First*, do microglia exist in functional clusters? It is still unclear whether transcriptional heterogeneity implicates functional specialization. Future studies are warranted to establish links between cell states and function. It is also unclear whether cell states that microglia assume in disease are beneficial or detrimental for neuronal and brain homeostasis. Gaining a deeper insight into how microglial clusters alter according to functional changes in health and disease will be critical in developing specific targets in microglia to preserve synapses and neuronal function. Importantly, many of these functional attributes may be located at microglial processes; hence, it will be critical to dissect subcellular organization of transcripts as in neurons. Taking into account that microglia likely lose processes as well as physiological profiles during homogenization and cell isolation protocols, in situ sequencing techniques will be key to reveal important and highly regulated subcellular information. *Second,* various microglial cell states are found throughout life, but it is unclear whether these are different subpopulations or whether they belong to one population of microglia that transitions between cell states in a spatiotemporal context. In line, it remains unanswered as to what extent microglial diversity is dictated by origin versus environment. This is highly relevant for therapeutic purposes to determine whether strategies should be focused on cellular origins and intrinsic features or on better understanding of how the environmental niche imprints microglial transcriptomes and their corresponding epigenetic landscape. *Finally*, how can we translate findings in mouse microglia to humans? (Böttcher et al., 2018; Geirsdottir et al., 2019; Masuda et al., 2019; Mathys et al., 2019; Sala Frigerio et al., 2019; Zhou et al., 2020) Latest data indicate limited convergence between murine and human microglia, in particular to candidate AD risk genes. One innovative approach to address this conundrum was introduced by two independent groups where they created “chimeric mice,” engrafting human inducible pluripotent stem cell‐ or embryonic stem cell‐derived microglia in brains of immunodeficient mice (Hasselmann et al., 2019; Mancuso et al., 2019). These chimeric models will be a powerful tool to understand how human microglia differ from mouse microglia in vivo and contribution of the brain region‐dependent microenvironment.

Region‐specific vulnerability is a key hallmark across neurologic disorders. Hence, spatiotemporal resolution of microglial cell states and insight into their functional relevance in neuronal health and function will significantly advance efforts to identify targets for biomarker development and treatment in neurologic diseases.

## CONFLICT OF INTEREST

All authors declare no competing financial conflict or conflict of interest related to this project.
